# Author Correction: Diurnal temperature range as a key predictor of plants’ elevation ranges globally

**DOI:** 10.1038/s41467-024-45797-9

**Published:** 2024-02-20

**Authors:** Arnaud Gallou, Alistair S. Jump, Joshua S. Lynn, Richard Field, Severin D. H. Irl, Manuel J. Steinbauer, Carl Beierkuhnlein, Jan-Chang Chen, Chang-Hung Chou, Andreas Hemp, Yohannes Kidane, Christian König, Holger Kreft, Alireza Naqinezhad, Arkadiusz Nowak, Jan-Niklas Nuppenau, Panayiotis Trigas, Jonathan P. Price, Carl A. Roland, Andreas H. Schweiger, Patrick Weigelt, Suzette G. A. Flantua, John-Arvid Grytnes

**Affiliations:** 1 Department of Biological Sciences, University of Bergen, PO Box 7803, 5020 Bergen, Norway; 2 Biological and Environmental Sciences, Faculty of Natural Sciences, University of Stirling, FK9 4LA Scotland, UK; 3 Department of Biological Sciences, University of Bergen and Bjerknes Centre for Climate Research, Bergen, Norway; 4 Department of Earth and Environmental Sciences, The University of Manchester, Manchester, UK; 5 School of Geography, University of Nottingham, Nottingham, NG7 2RD UK; 6 Biogeography and Biodiversity Lab, Institute of Physical Geography, Goethe-University Frankfurt, Altenhöferallee 1, 60438 Frankfurt, Germany; 7 Bayreuth Center of Ecology and Environmental Research & Department of Sport Science, University of Bayreuth, 95447 Bayreuth, Germany; 8 Chair of Biogeography, University of Bayreuth, 95440 Bayreuth, Germany; 9 Department of Botany, University of Granada, Granada, Spain; 10 Department of Forestry, National Pingtung University of Science and Technology, Pingtung, Taiwan; 11 Institute of Plant and Microbial Biology, Academia Sinica, Taipei, Taiwan; 12 Department of Plant Systematics, University of Bayreuth, 95440 Bayreuth, Germany; 13 Biodiversity, Macroecology & Biogeography, University of Göttingen, Büsgenweg 1, 37077 Göttingen, Germany; 14 Centre of Biodiversity and Sustainable Land Use, University of Göttingen, Büsgenweg 1, 37077 Göttingen, Germany; 15 Department of Plant Biology, Faculty of Basic Sciences, University of Mazandaran, P.O. Box: 47416-95447, Babolsar, Iran; 16 Institute of Biology, University of Opole, Oleska St., 45-052 Opole, Poland; 17 PAS Botanical Garden - Center for Biodiversity Conservation in Powsin, Prawdziwka St. 2, 02-952 Warszawa, Poland; 18 Department of Ecology, Environment and Plant Science, Stockholm University, 106 91 Stockholm, Sweden; 19 Department of Crop Science, School of Plant Sciences, Agricultural University of Athens, Iera Odos 75, 11855 Athens, Greece; 20 Department of Geography, University of Hawaii, Hilo, Hawaii USA; 21 Denali National Park, 4175 Geist Road, Fairbanks, AK 99709 USA; 22 Institute of Landscape and Plant Ecology, Department of Plant Ecology, University of Hohenheim, Ottilie-Zeller-Weg 2, 70599 Stuttgart, Germany; 23 Campus-Institut Data Science, University of Göttingen, Göttingen, Germany

**Keywords:** Ecosystem ecology, Biogeography, Biodiversity

Correction to: *Nature Communications* 10.1038/s41467-023-43477-8, published online 30 November 2023

The original version of this Article contained errors in the Supplementary Information. The legend for Supplementary Fig. 8 incorrectly read “(**c**) using standardized elevation gradients of 2500 m and exclusion zones of 250m. Standardization was performed from upper elevations” The title for Supplementary Table 1 incorrectly read “each 44 mountain gradients used in the study”. The correct version states “each of the 44 mountain gradients used in the study”. The legend for Supplementary Table 1 incorrectly read “difference between the maximum and minimum observation”. The correct version states “difference between the highest and lowest elevation observation”. The HTML has been updated to include a corrected version of the Supplementary Information.

### Supplementary information

Original Supplementary Information

Updated Supplementary Information

