# Supplementary material for: Diurnal temperature range as a key predictor of plants’ elevation ranges globally
Source: Nat Commun. 2023 Nov 30;14:7890. doi: 10.1038/s41467-023-43477-8 (PMC10689480; doi:10.1038/s41467-023-43477-8)
Supplement: Supplementary file 3 — Description of Additional Supplementary Files [file 41467_2023_43477_MOESM3_ESM.pdf]

Description of Additional Supplementary Files

Supplementary Data 1: Elevation range and climate data to reproduce all the analyses.

Supplementary Data 2: Climate data along elevation.
