# Supplementary material for: Diurnal temperature range as a key predictor of plants’ elevation ranges globally
Source: Nat Commun. 2023 Nov 30;14:7890. doi: 10.1038/s41467-023-43477-8 (PMC10689480; doi:10.1038/s41467-023-43477-8)
Supplement: Supplementary file 5 — Reporting Summary [file 41467_2023_43477_MOESM5_ESM.pdf]

nature portfolio

Corresponding author(s): Arnaud Gallou

Last updated by author(s): Oct 18, 2023

Reporting Summary

Nature Portfolio wishes to improve the reproducibility of the work that we publish. This form provides structure for consistency and transparency in reporting. For further information on Nature Portfolio policies, see our Editorial Policies and the Editorial Policy Checklist.

Statistics

For all statistical analyses, confirm that the following items are present in the figure legend, table legend, main text, or Methods section.

n/a	Confirmed	
☐	☒	The exact sample size (*n*) for each experimental group/condition, given as a discrete number and unit of measurement
☐	☒	A statement on whether measurements were taken from distinct samples or whether the same sample was measured repeatedly
☐	☒	The statistical test(s) used AND whether they are one- or two-sided<br>*Only common tests should be described solely by name; describe more complex techniques in the Methods section.*
☐	☒	A description of all covariates tested
☐	☒	A description of any assumptions or corrections, such as tests of normality and adjustment for multiple comparisons
☐	☒	A full description of the statistical parameters including central tendency (e.g. means) or other basic estimates (e.g. regression coefficient) AND variation (e.g. standard deviation) or associated estimates of uncertainty (e.g. confidence intervals)
☒	☐	For null hypothesis testing, the test statistic (e.g. *F*, *t*, *r*) with confidence intervals, effect sizes, degrees of freedom and *P* value noted<br>*Give P values as exact values whenever suitable.*
☐	☒	For Bayesian analysis, information on the choice of priors and Markov chain Monte Carlo settings
☐	☒	For hierarchical and complex designs, identification of the appropriate level for tests and full reporting of outcomes
☒	☐	Estimates of effect sizes (e.g. Cohen's *d*, Pearson's *r*), indicating how they were calculated

Our web collection on statistics for biologists contains articles on many of the points above.

Software and code

Policy information about availability of computer code

Data collection	Some data were extracted from the literature using a script (rsparser.py) written by myself which is available on my GitHub account (link in the paper)
Data analysis	All the analyses were performed in R (version 4.0.2)

For manuscripts utilizing custom algorithms or software that are central to the research but not yet described in published literature, software must be made available to editors and reviewers. We strongly encourage code deposition in a community repository (e.g. GitHub). See the Nature Portfolio guidelines for submitting code & software for further information.

Data

Policy information about availability of data

All manuscripts must include a data availability statement. This statement should provide the following information, where applicable:

- Accession codes, unique identifiers, or web links for publicly available datasets
- A description of any restrictions on data availability
- For clinical datasets or third party data, please ensure that the statement adheres to our policy

All the code to reproduce the results in the study is currently available on GitHub (link in the study). The dataset is currently available with some anonymized species names which might be deanonymized after acceptance of the manuscript.

Human research participants

Policy information about studies involving human research participants and Sex and Gender in Research.

Reporting on sex and gender	*Use the terms sex (biological attribute) and gender (shaped by social and cultural circumstances) carefully in order to avoid confusing both terms. Indicate if findings apply to only one sex or gender; describe whether sex and gender were considered in study design whether sex and/or gender was determined based on self-reporting or assigned and methods used. Provide in the source data disaggregated sex and gender data where this information has been collected, and consent has been obtained for sharing of individual-level data; provide overall numbers in this Reporting Summary. Please state if this information has not been collected. Report sex- and gender-based analyses where performed, justify reasons for lack of sex- and gender-based analysis.*
Population characteristics	*Describe the covariate-relevant population characteristics of the human research participants (e.g. age, genotypic information, past and current diagnosis and treatment categories). If you filled out the behavioural & social sciences study design questions and have nothing to add here, write "See above."*
Recruitment	*Describe how participants were recruited. Outline any potential self-selection bias or other biases that may be present and how these are likely to impact results.*
Ethics oversight	*Identify the organization(s) that approved the study protocol.*

Note that full information on the approval of the study protocol must also be provided in the manuscript.

Field-specific reporting

Please select the one below that is the best fit for your research. If you are not sure, read the appropriate sections before making your selection.

☐ Life sciences ☐ Behavioural & social sciences ☒ Ecological, evolutionary & environmental sciences

For a reference copy of the document with all sections, see nature.com/documents/nr-reporting-summary-flat.pdf

Ecological, evolutionary & environmental sciences study design

All studies must disclose on these points even when the disclosure is negative.

Study description	Decreasing elevational range sizes of plants with greater diurnal temperature range supports a novel biodiversity hypothesis and indicates elevated extinction risk of continental species.
Research sample	The data used in the study come from more than 50 sources from the literature, online sources or existing datasets, which are listed in the appendix.
Sampling strategy	We used all relevant data (vascular plant data with elevational range from mountain regions) available for our study, worldwide. In total, we collected more than 96,000 species' elevational ranges in 56 mountain regions.
Data collection	Data from the literature or online sources were extracted by myself, J-N.N., C.K., H.K., M.J.S., Y.K. as well as Cathy Jenks and Marten Winter. On site data were collected by A.N., A.N., C.A.R., J-C.C., J.P.P. and P.T., with the help of Kirsten O'Sullivan, Chi-Hua Chang and and the Forest Management Laboratory at the National Pingtung University of Science and Technology for the Taiwan data.
Timing and spatial scale	Data comes from various mountains around the globe.
Data exclusions	Some data were excluded due to poor sampling effort (dataset with more than 25% of species observed once) or lack of precision (elevational range given for a broader region than the sampled area). We removed a total of 38 observations with obviously incorrect elevation values, e.g. species with a minimum elevation greater than the maximum elevation or with an elevation higher than 6500 m, corresponding to the highest elevation recorded for vascular plants. These erroneous data were present in the original source and probably resulted from typographic errors, such as additional digits in elevation values.
Reproducibility	All data and code to reproduce the results are available.
Randomization	Not applicable.
Blinding	Not applicable.

Did the study involve field work? ☐ Yes ☒ No

Reporting for specific materials, systems and methods

We require information from authors about some types of materials, experimental systems and methods used in many studies. Here, indicate whether each material, system or method listed is relevant to your study. If you are not sure if a list item applies to your research, read the appropriate section before selecting a response.

Materials & experimental systems

n/a	Involved in the study
☒	☐ Antibodies
☒	☐ Eukaryotic cell lines
☒	☐ Palaeontology and archaeology
☒	☐ Animals and other organisms
☒	☐ Clinical data
☒	☐ Dual use research of concern

Methods

n/a	Involved in the study
☒	☐ ChIP-seq
☒	☐ Flow cytometry
☒	☐ MRI-based neuroimaging
