# Supplementary material for: Author Correction: Diurnal temperature range as a key predictor of plants’ elevation ranges globally
Source: Nat Commun. 2024 Feb 20;15:1554. doi: 10.1038/s41467-024-45797-9 (PMC10879124; doi:10.1038/s41467-024-45797-9)
Supplement: Supplementary file 1 — Original Supplementary Information [file 41467_2024_45797_MOESM1_ESM.pdf]

Supplementary Information

Diurnal temperature range as a key predictor of plants' elevation ranges globally

Arnaud Gallou, Alistair S. Jump, Joshua S. Lynn, Richard Field, Severin D.H. Irl, Manuel J. Steinbauer, Carl Beierkuhnlein, Jan-Chang Chen, Chang-Hung Chou, Andreas Hemp, Yohannes Kidane, Christian König, Holger Kreft, Alireza Naqinezhad, Arkadiusz Nowak, Jan-Niklas Nuppenau, Panayiotis Trigas, Jonathan P. Price, Carl A. Roland, Andreas H. Schweiger, Patrick Weigelt, Suzette G.A. Flantua, John-Arvid Grytnes

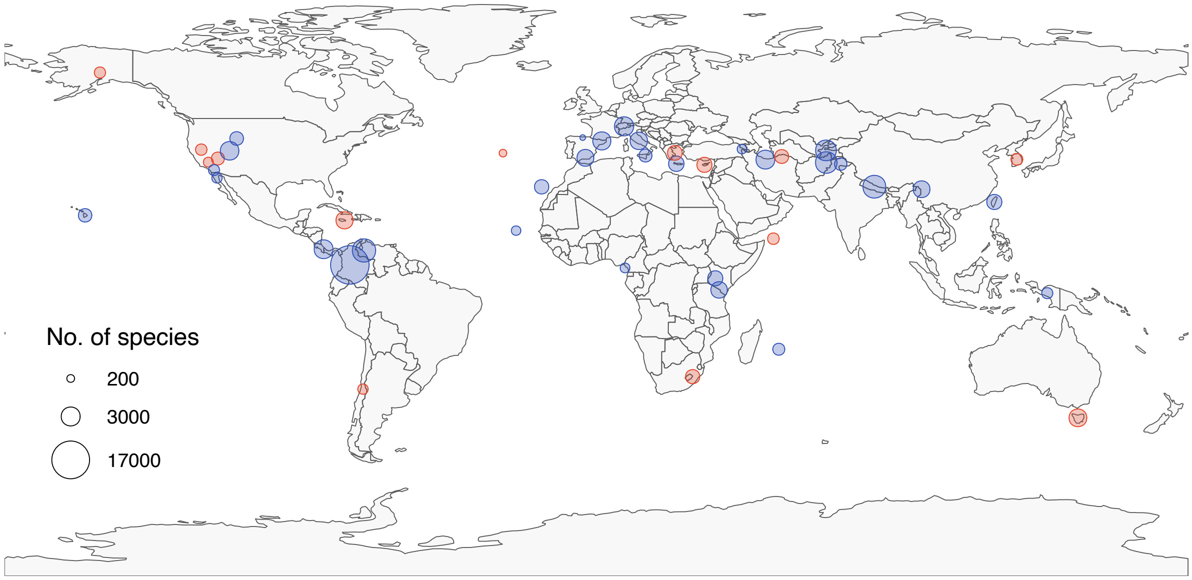

Supplementary Figure 1. Spatial distribution of the 44 mountains used in the study. Colored points indicate elevation gradients < 2500 m (red) and elevation gradients ≥ 2500 m (blue). The map was created in R (version 4.0.2)[1] using the ggplot2 (version 3.4.1)[2] and rnaturalearth (version 0.1.0)[3] packages.

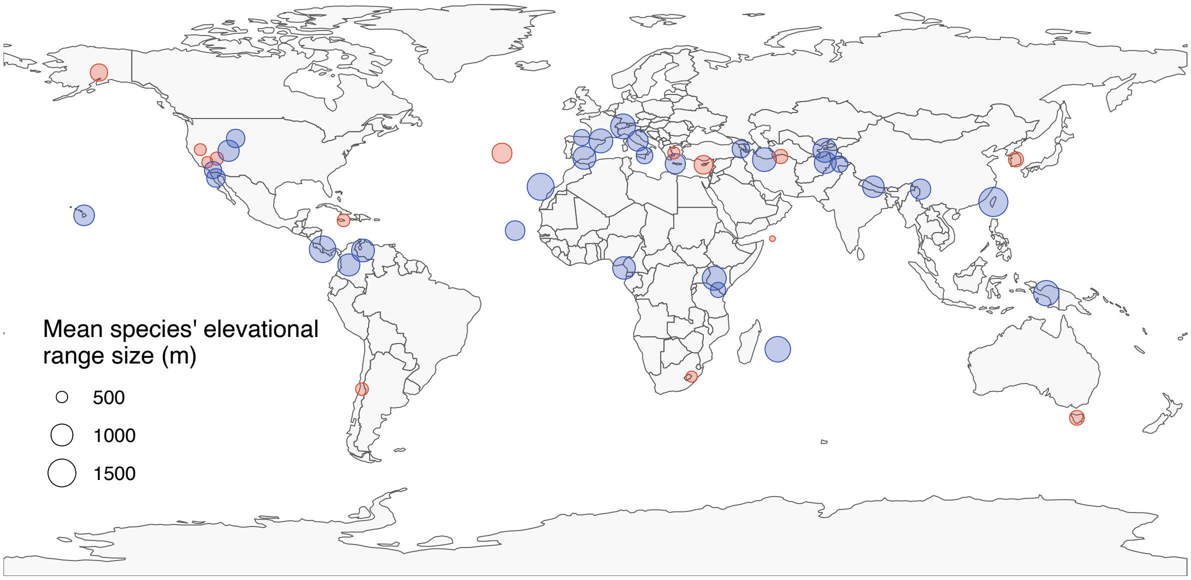

Supplementary Figure 2. Mean species' elevation ranges in each mountain used in the study. Colored points indicate elevation gradients < 2500 m (red) and elevation gradients ≥ 2500 m (blue). The map was created in R (version 4.0.2)[1] using the ggplot2 (version 3.4.1)[2] and rnaturalearth (version 0.1.0)[3] packages.

Supplementary Table 1. Details about each 44 mountain gradients used in the study. The elevation span is the difference between the maximum and minimum observation in a given mountain. The number of species is the number of species after naming homogenization.

Location	Alpha-3 ISO	Elevation span (m)	No. of species	Reference
Afghanistan	AFG	5520	4274	(*4*, *5*)
Alborz Mts.	IRN	4800	2973	(*6*, *7*)
Azores	PRT	2350	192	(*8*)
Baekdudaegan Mts.	KOR	1500	746	(*9*)
Bioko	GNQ	2800	404	(*10*)
Canary	ESP	3540	1345	(*11*)
Cantabria	ESP	2600	129	(*12*)
Cape Verde	CPV	2800	365	(*13*, *14*)
Chicauma	CHL	1700	487	(*15*)
Colombian Andes	COL	5000	17764	(*16*)
Crete	GRC	2500	1693	(*17–19*)
Cyprus	CYP	2000	1582	(*20*, *21*)
Denali	USA	1660	626	(*22*)
Drakensberg	ZAF, LSO	1500	1312	(*23*)
Euboea	GRC	1700	1451	(*24–26*)
Golestan	IRN	2000	1229	(*27*)
Hawaii	USA	4110	1113	(*28*)
Hengduan	CHN	5250	2142	(*29*, *30*)
Jamaica	JAM	2280	2263	(*31*)
Jaya	IDN	4750	608	(*32*)
La Amistad	PAN, CRI	4000	2973	(*33*)
Lazio	ITA	2600	2389	(*34*)
Mt. Ararat	TUR	3300	368	(*35*)
Mt. Etna	ITA	3100	993	(*36*)
Mt. Kilimanjaro	TZA	4070	2033	(*37*)
Nanga Parbat	PAK	4300	876	(*38*)
Nepal	NPL	6430	4907	(*39–41*)
Nevada Test Site	USA	2240	987	(*42*)
Owens Peak	USA	1900	418	(*43*)
Reunion	REU	3060	778	(*44–46*)
Santa Rosa Mts.	USA	2690	592	(*47*)
Sierra Nevada	ESP	3200	2177	(*48*)
Sierra San Pedro Martir	MEX	2940	478	(*49*)
Socotra	YEM	1550	689	(*50*)
South-Eastern Pyrenees	ESP	3400	2657	(*51*, *52*)
Swiss Alps	CHE	4310	3100	(*53*)
Tahoe	USA	1950	663	(*54*)
Taiwan	TWN	3990	1587	(*55*)
Tajikistan	TJK	5500	3830	(*56*)
Tasmania	AUS	1600	2520	(*57*)
Utah	USA	4230	2846	(*58*)
Venezuelan Andes	VEN	5100	5176	(*59*)
Western Kenya	KEN	5310	1565	(*60*, *61*)
Wind River Mts.	USA	2740	1164	(*62*)

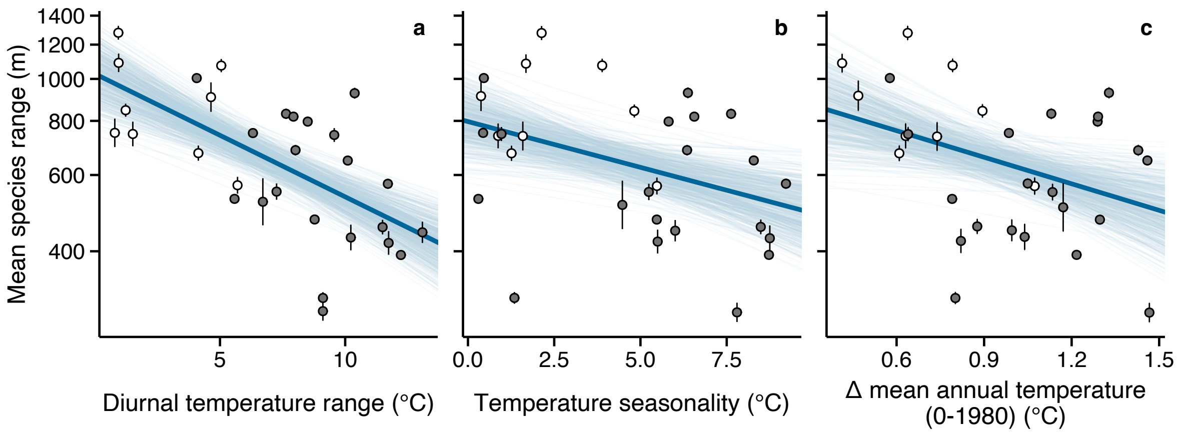

Supplementary Figure 3. Response of mean species' elevation ranges to diurnal temperature range (a), temperature seasonality (b) and the variation of mean annual temperature from 0 to 1980 AD (c) using standardized elevation gradients from lower elevations. Points represent the estimated mean elevation ranges with their respective standard error in each of the 30 standardized elevation gradients ≥ 2500 m in length. Thick blue lines are the posterior mean calculated from 600 random draws sampled from the 95% credible interval (thin blue lines). Colored dots indicate island (white) and continental (dark gray) mountains. Source data are provided as a Source Data file.

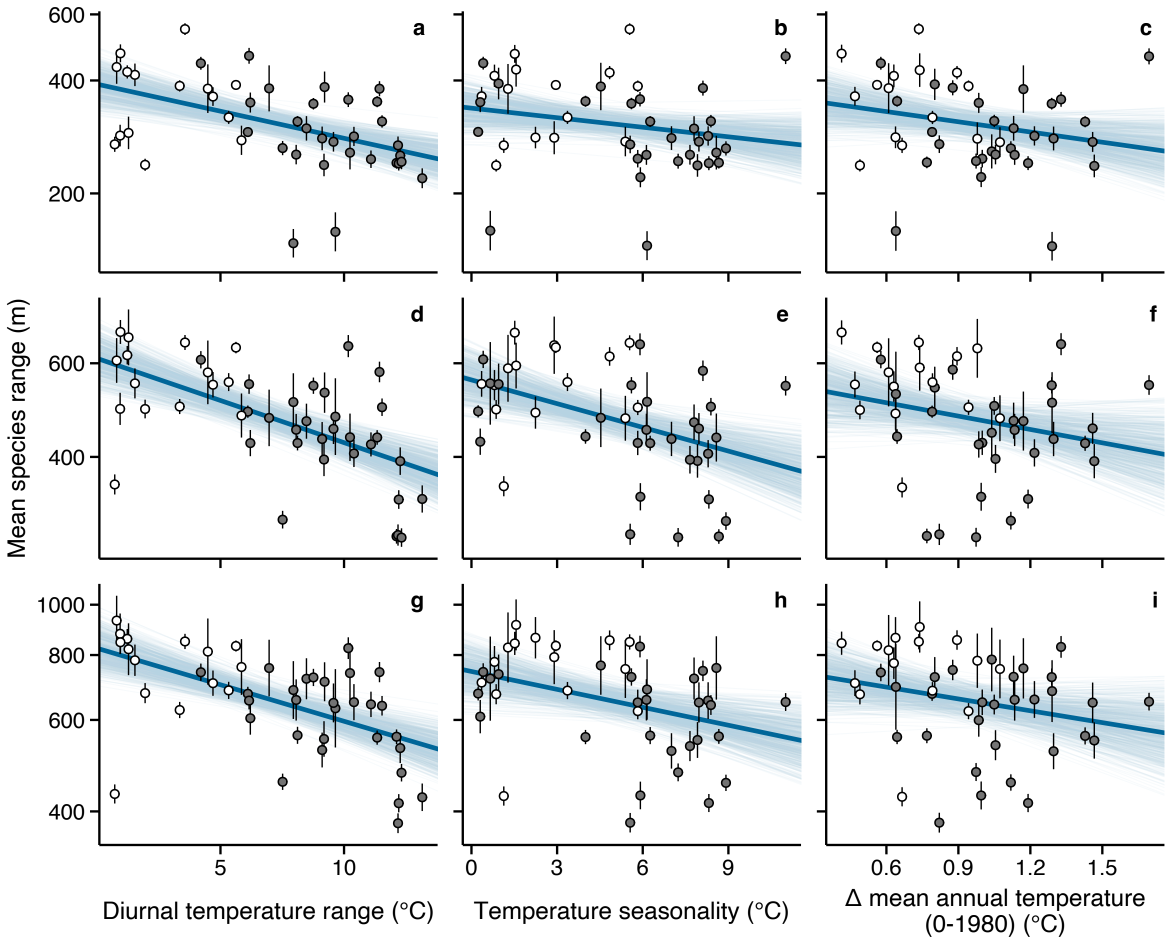

Supplementary Figure 4. Response of mean species' elevation ranges to diurnal temperature range, temperature seasonality and the variation of mean annual temperature from 0 to 1980 AD using a standardized elevation span of 1500 m. The different panels show the responses of species' range sizes to each temperature variation predictor using all species (**a-c**) and after discarding species found exclusively in the upper and lower 250 m (**a-f**) and 500 m (**g-i**) of the elevation gradient. Thick blue lines are the posterior mean calculated from 600 random draws sampled from the 95% credible interval (thin blue lines). Colored dots indicate island (white) and continental (dark gray) mountains. Each elevation gradient length was standardized from upper elevations. Source data are provided as a Source Data file.

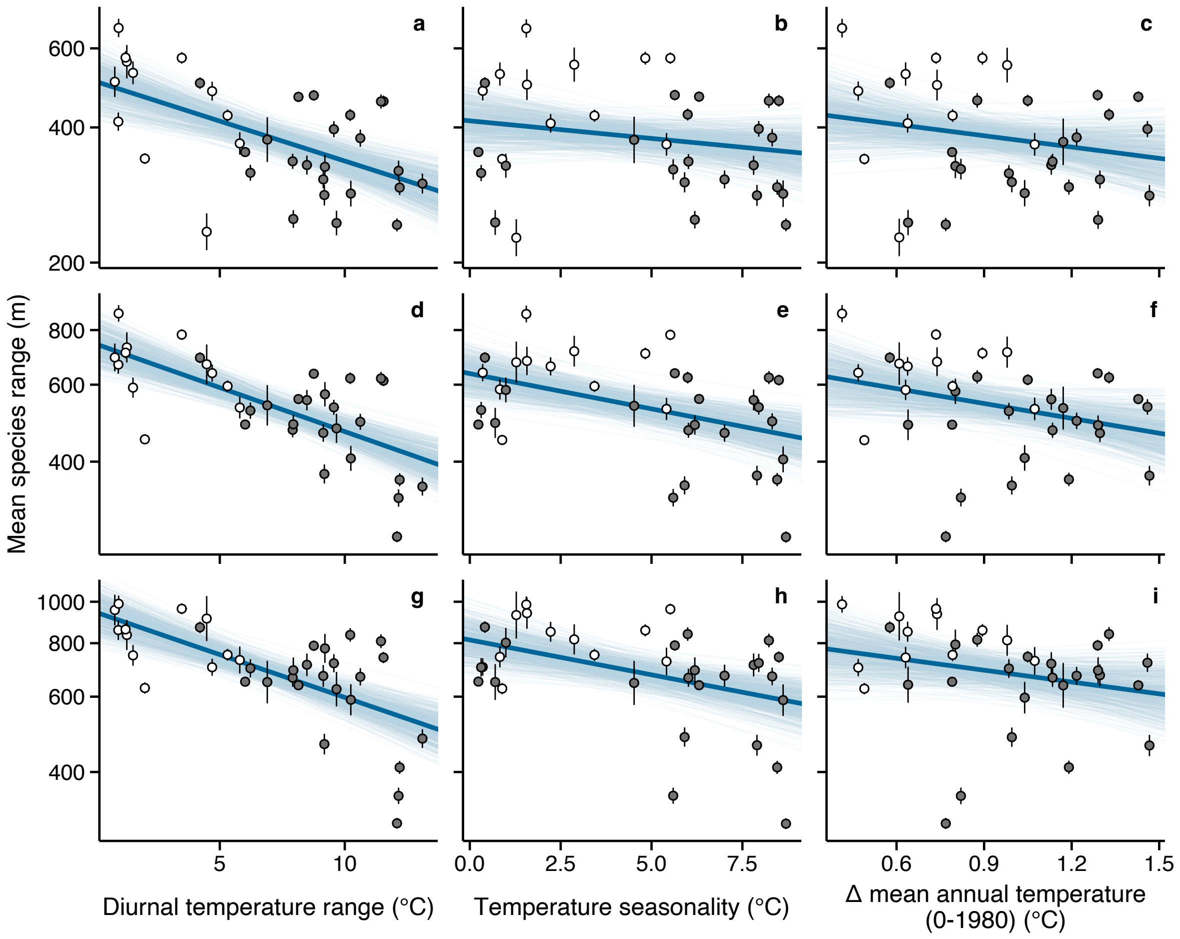

Supplementary Figure 5. Response of mean species' elevation ranges to diurnal temperature range, temperature seasonality and the variation of mean annual temperature from 0 to 1980 AD using a standardized elevation span of 2000 m. The different panels show the responses of species' range sizes to each temperature variation predictor using all species (**a-c**) and after discarding species found exclusively in the upper and lower 250 m (**a-f**) and 500 m (**g-i**) of the elevation gradient. Thick blue lines are the posterior mean calculated from 600 random draws sampled from the 95% credible interval (thin blue lines). Colored dots indicate island (white) and continental (dark gray) mountains. Each elevation gradient length was standardized from upper elevations. Source data are provided as a Source Data file.

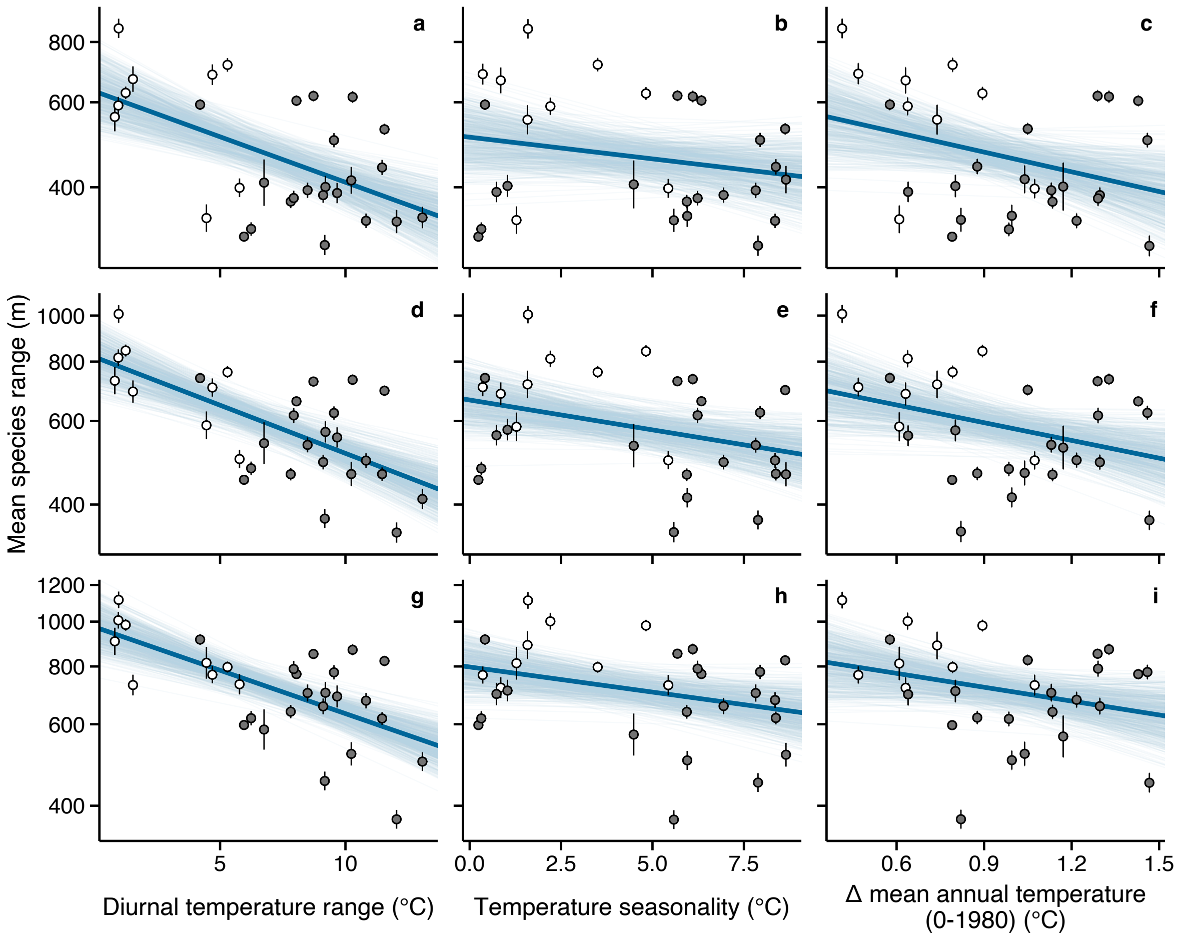

Supplementary Figure 6. Response of mean species' elevation ranges to diurnal temperature range, temperature seasonality and the variation of mean annual temperature from 0 to 1980 AD using a standardized elevation span of 2500 m. The different panels show the responses of species' range sizes to each temperature variation predictor using all species (**a-c**) and after discarding species found exclusively in the upper and lower 250 m (**d-f**) and 500 m (**g-i**) of the elevation gradient. Thick blue lines are the posterior mean calculated from 600 random draws sampled from the 95% credible interval (thin blue lines). Colored dots indicate island (white) and continental (dark gray) mountains. Each elevation gradient length was standardized from upper elevations. Source data are provided as a Source Data file.

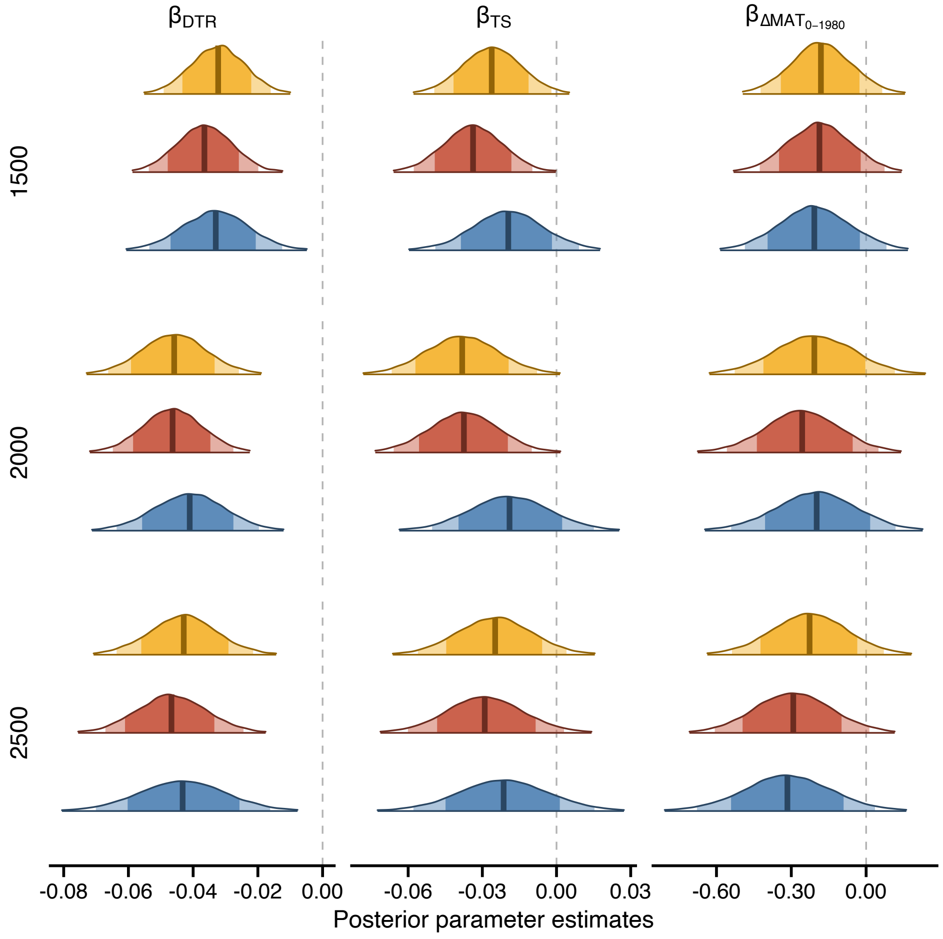

Supplementary Figure 7. Posterior distributions from the global-scale analyses for diurnal temperature range (DTR), temperature seasonality (TS) and the variation of mean annual temperature from 0 to 1980 AD ($\Delta MAT_{0\text{-}1980}$). The plots show the median values of the regression coefficients (vertical thick lines) with the 80% (dark shading), 95% (light shading) and 99% (outlines) credible intervals for standardized elevation gradient length of 1500, 2000 and 2500 m. Colors indicate analyses with exclusion zones of 500 m (yellow), 250 m (red) and 0 m (blue).

Supplementary Table 2. Results of the model selection. ΔWAIC and ΔLOO are the relative difference in information criterion between a given and the best model. WAIC and LOO values are from models using standardized gradient length (from upper elevations) and exclusion zones of 2500 m and 250 m, respectively. DTR: diurnal temperature range; TS: temperature seasonality; MAT: mean annual temperature; AP: annual precipitation; $\Delta MAT_{0\text{-}1980}$: temperature variation from 0 to 1980 AD; $MAP_{0\text{-}1980}$: mean annual precipitation from 0 to 1980 AD.

Model	WAIC	ΔWAIC	LOOIC	ΔLOOIC
DTR	-6.37	0.00	-6.18	0.00
DTR * MAT	-3.73	2.63	-3.12	3.05
DTR * land type	-3.16	3.21	-2.57	3.60
DTR * AP	-3.02	3.35	-2.51	3.66
ΔMAT_{0-1980} * land type	0.86	7.22	1.78	7.96
TS * land type	2.40	8.76	3.10	9.28
TS	5.73	12.09	5.87	12.05
ΔMAT_{0-1980}	5.74	12.11	5.91	12.09
ΔMAT_{0-1980} * MAP_{0-1980}	9.10	15.47	9.70	15.88

Supplementary Table 3. Statistical details of the global-scale analyses. Multiple values in the $P(\beta < 0)$ column are the $P(\beta < 0)$ of the first, second and the interaction of the two predictors, respectively. DTR: diurnal temperature range; TS: temperature seasonality; MAT: mean annual temperature; AP: annual precipitation; $\Delta MAT_{0\text{-}1980}$: temperature variation from 0 to 1980 AD; $MAP_{0\text{-}1980}$: mean annual precipitation from 0 to 1980 AD. Statistics are from models using standardized elevation gradients from upper elevations.

Model	Elevation span (m)	Exclusion zone (m)	R^2	Standard error	$P(\beta < 0)$
DTR	1500	0	0.22	0.10	1
ΔMAT_{0-1980}	1500	0	0.05	0.06	0.92
TS	1500	0	0.05	0.06	0.91
DTR	1500	250	0.35	0.10	1
ΔMAT_{0-1980}	1500	250	0.06	0.07	0.93
TS	1500	250	0.19	0.10	1
DTR	1500	500	0.32	0.11	1
ΔMAT_{0-1980}	1500	500	0.06	0.07	0.93
TS	1500	500	0.13	0.09	0.98
DTR	2000	0	0.32	0.11	1
ΔMAT_{0-1980}	2000	0	0.05	0.07	0.89
TS	2000	0	0.04	0.07	0.88
DTR	2000	250	0.47	0.10	1
ΔMAT_{0-1980}	2000	250	0.09	0.08	0.95
TS	2000	250	0.19	0.10	0.99
DTR	2000	500	0.42	0.11	1
ΔMAT_{0-1980}	2000	500	0.05	0.07	0.9
TS	2000	500	0.18	0.10	0.99
DTR	2500	0	0.29	0.12	1
ΔMAT_{0-1980}	2500	0	0.11	0.09	0.96
TS	2500	0	0.05	0.07	0.88
DTR	2500	250	0.43	0.11	1
ΔMAT_{0-1980}	2500	250	0.12	0.10	0.97
TS	2500	250	0.12	0.09	0.96
DTR	2500	500	0.41	0.11	1
ΔMAT_{0-1980}	2500	500	0.08	0.09	0.93
TS	2500	500	0.10	0.09	0.95
DTR * AP	2500	250	0.44	0.10	0.99; 0.29; 0.56
DTR * land type	2500	250	0.46	0.10	0.91; 0.17; 0.82
DTR * MAT	2500	250	0.46	0.10	0.77; 0.15; 0.87
ΔMAT_{0-1980} * land type	2500	250	0.39	0.11	0.27; 0.01; 0.93

(continued)

Model	Elevation span (m)	Exclusion zone (m)	R^2	Standard error	$P(\beta < 0)$
ΔMAT$_{0-1980}$ * MAP$_{0-1980}$	2500	250	0.19	0.09	0.76; 0.25; 0.73
TS * land type	2500	250	0.35	0.11	0.64; 0.03; 0.65

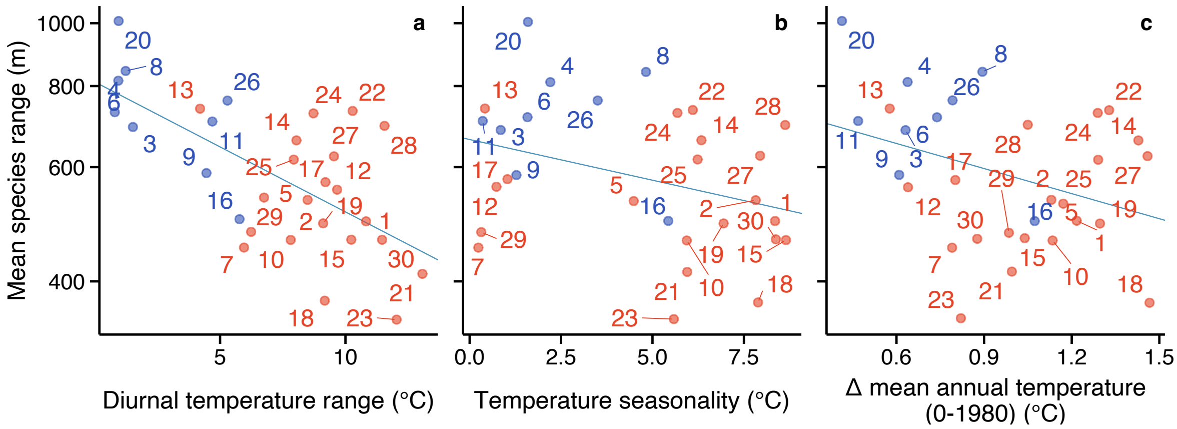

Supplementary Figure 8. Names of the elevation gradients as shown in Fig. 2. Response of mean species' elevation ranges to diurnal temperature range (**a**), temperature seasonality (**b**) and the variation of mean annual temperature from 0 to 1980 AD (**c**) using standardized elevation gradients from lower elevations. Colors indicate continental (orange) and island (blue) mountains. 1: Afghanistan; 2: Alborz Mts.; 3: Bioko; 4: Canary; 5: Cantabria; 6: Cape Verde; 7: Colombian Andes; 8: Crete; 9: Hawaii; 10: Hengduan; 11: Jaya; 12: Western Kenya; 13: La Amistad; 14: Lazio; 15: Mt. Ararat; 16: Mt. Etna; 17: Mt. Kilimanjaro; 18: Nanga Parbat; 19: Nepal; 20: Reunion; 21: Santa Rosa Mts.; 22: Sierra Nevada (Spain); 23: Sierra San Pedro Martir; 24: South-Eastern Pyrenees; 25: Swiss Alps; 26: Taiwan; 27: Tajikistan; 28: Utah; 29: Venezuelan Andes; 30: Wind River Mts.

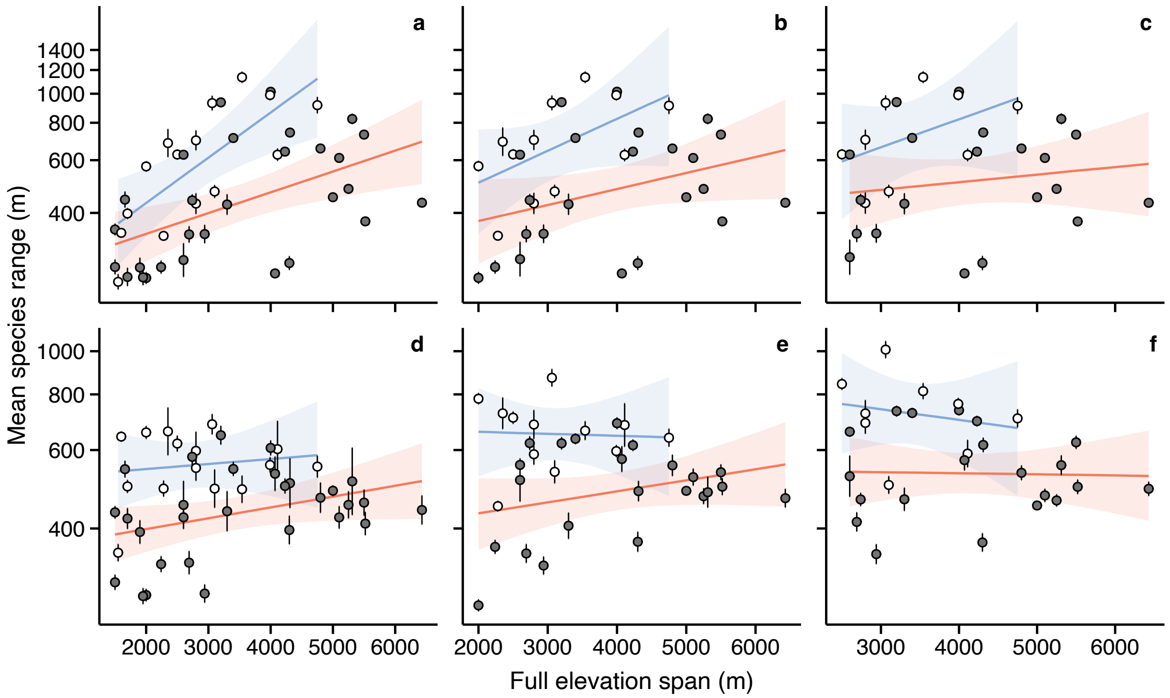

Supplementary Figure 9. Response of species' range sizes to full elevation spans (difference between the highest and lowest observation in any given mountain) in island and continental mountains. Top panels show the influence of full elevation spans on species' range sizes estimated from models using no standardization and no exclusion zones for elevation spans ≥ 1500 m (**a**), ≥ 2000 m (**b**) and ≥ 2500 m (**c**). Bottom panels show the influence of full elevation spans on species' range sizes estimated from models using standardized elevation gradients of 1500 m (**d**), 2000 m (**e**) and 2500 m (**f**) and exclusion zones of 250 m. Standardization was performed from upper elevations. These plots show that the standardization minimized the influence of elevation spans and effectively accounted for mountain heights. Island mountains (white dots, blue regressions) have larger mean species' ranges than continental mountains (dark gray dots, orange regressions) due to different short-term temperature variation. Source data are provided as a Source Data file.

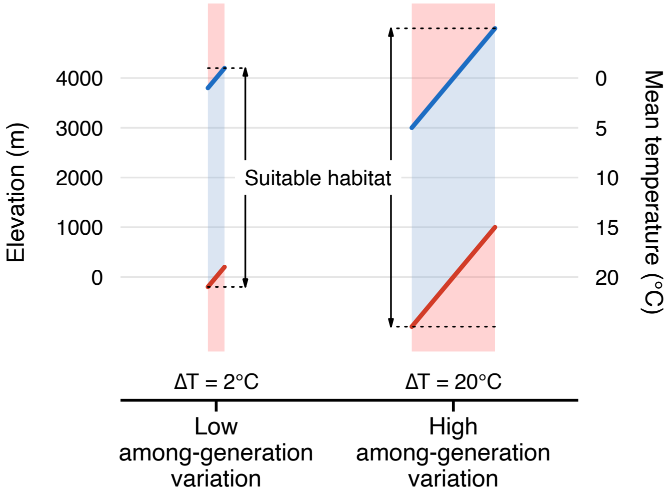

Supplementary Figure 10. Illustration of Gilchrist's hypothesis in mountains with low within-generation variation but variable among-generation variation. Assuming that individuals can survive any temperatures, Gilchrist's hypothesis links the reproductive success of individuals to performance breadth (i.e. the suitable temperatures for a population to produce the next generation; represented here by the shaded blue areas). Shaded red areas represent elevations where individuals can survive but cannot reproduce due to unfavorable conditions, thereby being unable to sustain a population over successive generations. Under such assumptions, and in the absence of other factors, a species will occupy the full range of temperatures corresponding to its performance breadth.

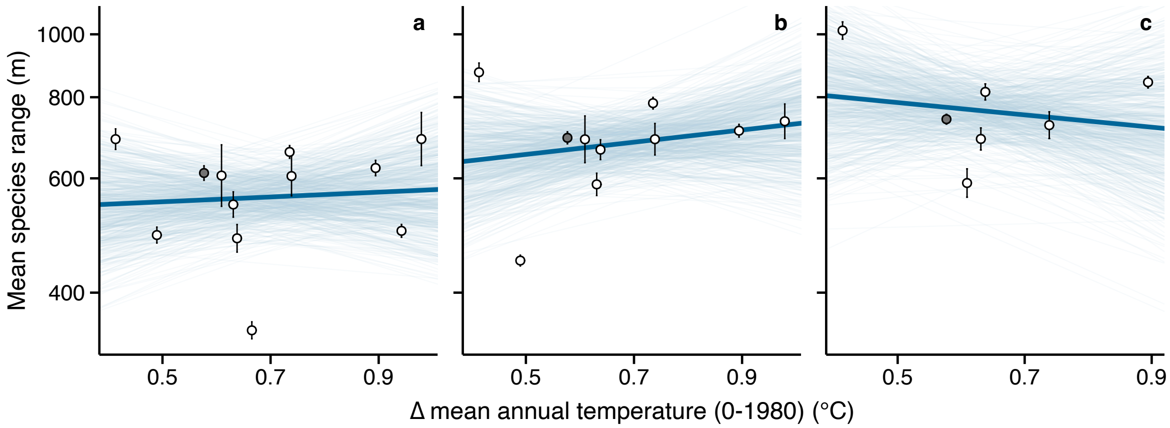

Supplementary Figure 11. Response of mean species' elevation ranges to the variation of mean annual temperature from 0 to 1980 AD for elevation gradients with low within-generation variation (lowest third of the diurnal temperature range gradient). Settings of these analyses are similar to that of the first column of Fig. 4 in Gilchrist[63], which shows that the influence of among-generation variation on species' performance breadth is stronger when within-generation variation is low. The different panels show the responses of species' range sizes for standardized elevation gradients of 1500 m (**a**), 2000 m (**b**) and 2500 m (**c**) with exclusion zones of 250 m. Thick lines are the posterior mean calculated from 600 random draws sampled from the 95% credible interval (thin blue lines). Colored dots indicate island (white) and continental (dark gray) mountains. Each elevation gradient was standardized from upper elevations. Source data are provided as a Source Data file.

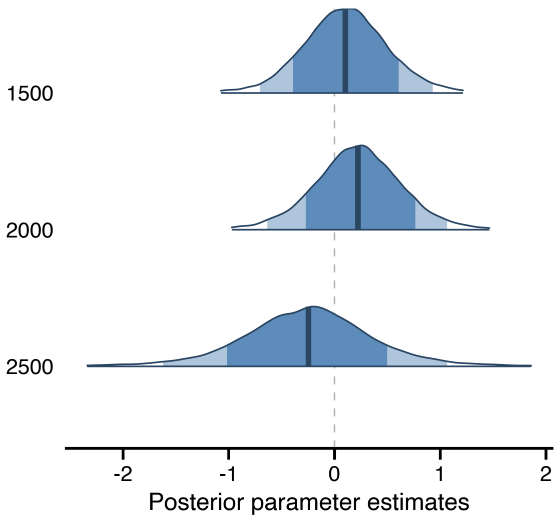

Supplementary Figure 12. Posterior distributions from the variation of mean annual temperature from 0 to 1980 AD for mountain gradients with low within-generation variation (lowest third of the diurnal temperature range gradient). The plots show the median values of the regression coefficients (vertical thick lines) with the 80% (dark shading), 95% (light shading) and 99% (outlines) credible intervals for standardized elevation gradient length of 1500, 2000 and 2500 m. Dashed line indicates 0.

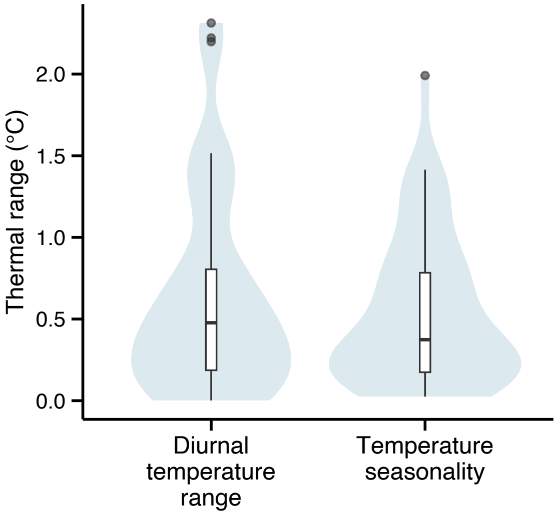

Supplementary Figure 13. Range of thermal variability within mountains. The thermal range corresponds to the difference between the maximum and minimum values for diurnal temperature range and temperature seasonality within each elevation gradient.

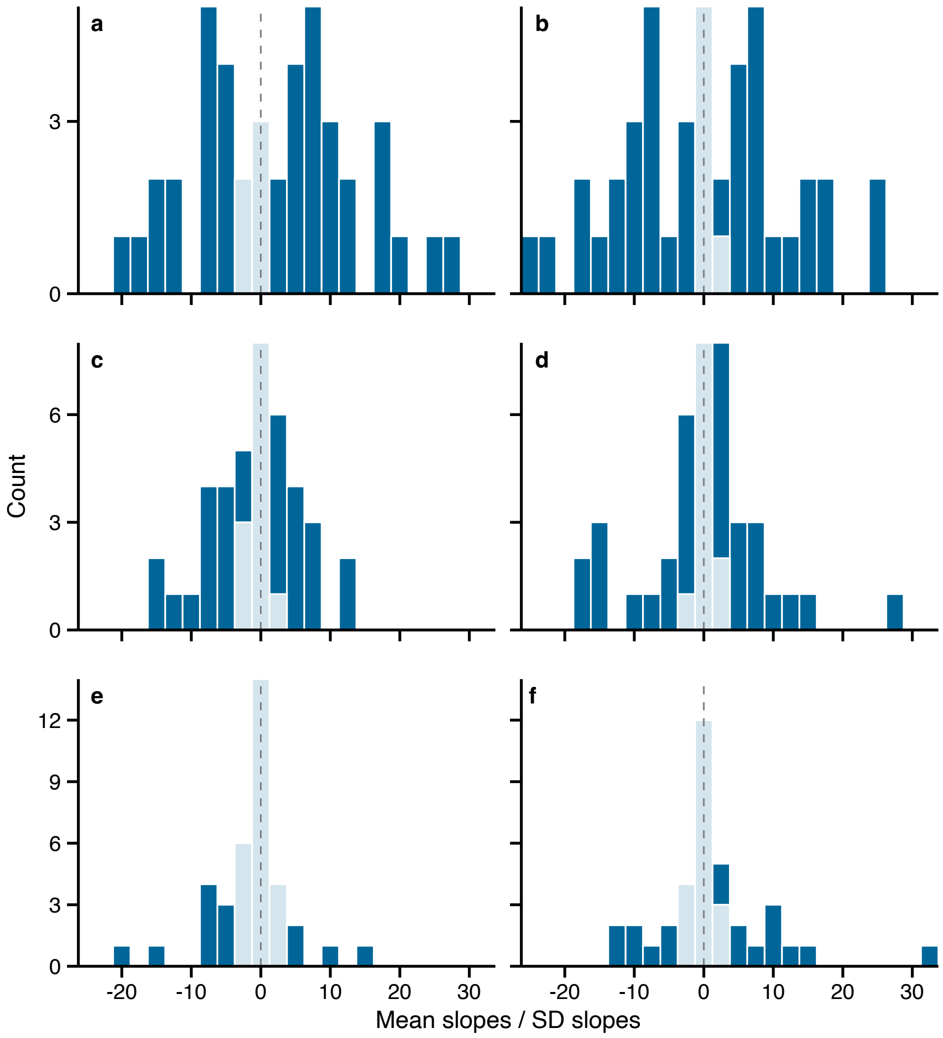

Supplementary Figure 14. Responses of species' elevation ranges to diurnal temperature range (a, c, e) and temperature seasonality (b, d, f) within each 44 mountains used in the study. The different panels indicate analyses performed using all species (**a-b**) and after discarding species found exclusively in the upper and lower 250 m (**c-d**) and 500 m (**e-f**) of the elevation gradients. Histograms are of mean slope terms divided by their standard deviation from the 95% credible interval for the local-scale analyses. Colors indicate whether the 95% credible interval includes 0 (light blue) or not (dark blue). Dashed lines indicate 0. Source data are provided as a Source Data file.

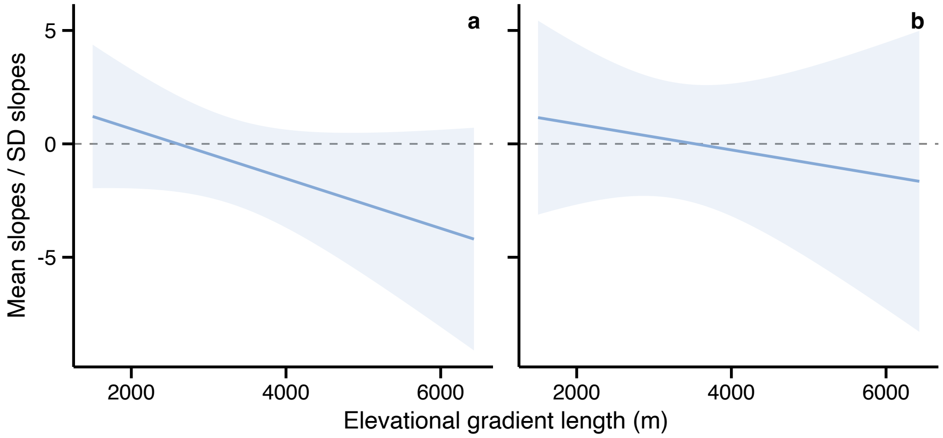

Supplementary Figure 15. Influence of elevation gradient length on slope direction within mountains for temperature seasonality (a) and diurnal temperature range (b).

Supplementary Table 4. Ratio of positive and negative slope estimates (%) for the local-scale analyses.

Model	Exclusion zone (m)	Positive	Negative	Positive low uncertainties	Negative low uncertainties	Positive high uncertainties	Negative high uncertainties
DTR	0	50	50	50	39	0	11
DTR	250	45	55	34	39	11	16
DTR	500	34	66	16	30	18	36
TS	0	52	48	43	43	9	5
TS	250	52	48	41	34	11	14
TS	500	55	45	32	25	23	20

Supplementary References

1. Team, R. C. R: a language and environment for statistical computing. (2020).

2. Wickham, H. *ggplot2: Elegant graphics for data analysis*. (Springer-Verlag New York, 2016).

3. South, A. *Rnaturalearth: World map data from natural earth*. (2017).

4. Breckle, S.-W., Hedge, I. C. & Rafiqpoor, M. D. *Vascular plants of Afghanistan: an augmented checklist*. (Scientia Bonnensis, Bonn, 2013).

5. Weigelt, P., König, C. & Kreft, H. GIFT: a Global Inventory of Floras and Traits for macroecology and biogeography. *Journal of Biogeography* **47**, 16–43 (2020).

6. Rechinger, K. H. *Flora Iranica*. vols. 1-181 (Natural History Museum, Vienna, 2005).

7. Assadi, M., Massoumi, A. A., Khatamsaz, M. & Mozaffarian, V. *Flora of Iran*. vols. 1-60 (Research Institute of Forest Publication, Tehran, 2008).

8. ATLANTIS 3.0 database.

9. Lee, C.-B., Chun, J.-H., Song, H.-K. & Cho, H.-J. Altitudinal patterns of plant species richness on the Baekdudaegan Mountains, South Korea: mid-domain effect, area, climate, and Rapoport's rule. *Ecological Research* **28**, 67–79 (2013).

10. Romero, A., Cabezas, F. & Velayos, M. Caldera de Luba scientific reserve: proposal of biodiversity assessment as a tool for biodiversity conservation in tropical areas. (2015).

11. Biocan | inicio.

12. Gómez, J. A. D. *Catálogo de la flora vascular de cantabria*. (Jolube Consultor Botánico y Editor, 2014).

13. Brochmann, C., Rustan, Ø. H., Lobin, W. & Kilian, N. The endemic vascular plants of the Cape Verde Islands, W Africa. *Sommerfeltia* **24**, 1–363 (1997).

14. Arechavaleta, M., Zurita, N., Marrero, M. C. & Martín, J. L. *Lista preliminar de especies silvestres de Cabo Verde: hongos, plantas y animales terrestres*. (Consejería de Medio Ambiente y Ordenación Territorial, Gobierno de Canarias, 2005).

15. García, N. Caracterización de la flora vascular de Altos de Chicauma, Chile (33º S). *Gayana. Botánica* **67**, 65–112 (2010).

16. Bernal, R., Gradstein, S. R. & Celis, M. *Catálogo de plantas y líquenes de colombia*. (Universidad Nacional de Colombia, Bogotá, 2019).

17. Turland, N. J., Chilton, L. & Press, J. R. *Flora of the Cretan area: annotated checklist & atlas*. (H.M. Stationery Office, 1993).

18. Jahn, R. & Schönfelder, P. *Exkursionsflora für Kreta*. (Ulmer, Stuttgart, 1995).

19. Strid, A. *Atlas of the Aegean flora*. (Botanic Garden; Botanical Museum Berlin, 2016).

20. Meikle, R. D. *Flora of Cyprus*. vol. 1 (The Bentham-Moxon Trust, Kew, 1977).

21. Meikle, R. D. *Flora of Cyprus*. vol. 2 (The Bentham-Moxon Trust, Kew, 1985).

22. Roland, C. A., Stehn, S. E. & Schmidt, J. H. Species richness of multiple functional groups peaks in alpine tundra in subarctic Alaska. *Ecosphere* **8**, e01848 (2017).

23. Hilliard, O. M. & Burtt, B. L. *The botany of the Southern Natal Drakensberg*. (National Botanic Gardens, Dublin, 1987).

24. Rechinger, K. H. *Die flora von Euboea*. (Botanische Jahrbücher für Systematik, Pflanzengeschichte und Pflanzengeographie, Stuttgart, 1961).

25. Trigas, P. & Iatrou, G. Additions to the flora of Evvia (Greece). *Botanika Chronika* **13**, 273–286 (2000).

26. Trigas, P. & Iatrou, G. The local endemic flora of Evvia (W Aegean, Greece). *Willdenowia* **36**, 257–270 (2006).

27. Akhani, H. *Plant biodiversity of Golestan national park, Iran*. (Biologiezentrum des O. O. Landesmuseums, Tehran, 1998).

28. Price, J. P. Floristic biogeography of the Hawaiian Islands: influences of area, environment and paleogeography. *Journal of Biogeography* **31**, 487–500 (2004).

29. Wang, W. T. *et al. Vascular plants of the Hengduan mountains*. vol. 1 (Science Press, Beijing, 1993).

30. Wang, W. T. *et al. Vascular plants of the Hengduan mountains*. vol. 2 (Science Press, Beijing, 1994).

31. Adams, C. D. *Flowering plants of Jamaica*. (University of the West Indies, Kingston, 1972).

32. Johns, R. J., Edwards, P. J., Utteridge, T. M. A. & Hopkins, H. C. F. *Guide to the alpine and sub-alpine flora of Mount Jaya*. (Royal Botanic Gardens, Kew, 2006).

33. Monro, A. *et al.* A first checklist to the vascular plants of La Amistad International Park (PILA), Costa Rica-Panama. *Phytotaxa* **322**, 1–283 (2017).

34. Anzalone, B., Iberite, M. & Lattanzi, E. La flora vascolare del Lazio. *Informatore Botanico Italiano* **42**, 187–317 (2010).

35. Zeynalov, Y. & Türkoğlu, M. *Ağrı dağı'nın florası*. (Iğdır Üniversitesi, Iğdır, 2015).

36. Sciandrello, S., Minissale, P. & Giusso del Galdo, G. Vascular plant species diversity of Mt. Etna (Sicily): endemicity, insularity and spatial patterns along the altitudinal gradient of the highest active volcano in Europe. *PeerJ* **8**, e9875 (2020).

37. Hemp, A. Vegetation database East Africa. *Biodiversity & Ecology* **4**, 292–292 (2012).

38. Dickoré, W. B. & Nüsser, M. Flora of Nanga Parbat (NW Himalaya, Pakistan): an annotated inventory of vascular plants with remarks on vegetation dynamics. *Englera* 3–253 (2000) doi:10.2307/3776769.

39. Kriechbaum, M. *Flora, vegetation und landnutzung des Muktinath-Tales (Mustang, Nepal) als beziehungsmuster von naturräumlicher ausstattung-und menschlicher gestaltung im zentralhimalaya*. (Dissertationes Botanicae, 2004).

40. Rokaya, M. B., Raskoti, B. B., Timsina, B. & Münzbergová, Z. An annotated checklist of the orchids of Nepal. *Nordic Journal of Botany* **31**, 511–550 (2013).

41. Annotated checklist of the flowering plants of Nepal. (2021).

42. Beatley, J. C. *Vascular plants of the Nevada Test Site and Central-Southern Nevada: ecologic and geographic distributions*. (1976).

43. Fraga, N. The vascular flora of the Owens Peak eastern watershed, Southern Sierra Nevada, California. *Aliso* **25**, 1–29 (2008).

44. Jacquemyn, H., Micheneau, C., Roberts, D. L. & Pailler, T. Elevational gradients of species diversity, breeding system and floral traits of orchid species on Réunion island. *Journal of Biogeography* **32**, 1751–1761 (2005).

45. Strasberg, D. *et al.* An assessment of habitat diversity and transformation on La Réunion island (Mascarene islands, Indian Ocean) as a basis for identifying broad-scale conservation priorities. *Biodiversity and Conservation* **14**, 3015–3032 (2005).

46. Conservatoire Botanique National - Centre Permanent d'Initiatives pour l'Environnement CBN-CPIE Mascarin.

47. Checklist of the vascular flora of Deep Canyon.

48. Lorite, J., Ros-Candeira, A., Alcaraz-Segura, D. & Salazar-Mendías, C. FloraSNevada: a trait database of the vascular flora of Sierra Nevada, Southeast Spain. *Ecology* **101**, e03091 (2020).

49. Thorne, R., Moran, R. & Minnich, R. Vascular plants of the high Sierra San Pedro Mártir, Baja California, Mexico: an annotated checklist. *Aliso* **28**, 1–50 (2010).

50. De Sanctis, M. *et al.* Classification and distribution patterns of plant communities on Socotra island, Yemen. *Applied Vegetation Science* **16**, 148–165 (2013).

51. Ortuño, E. C. i. & Sugrañes, J. M. N. i. *Flora i vegetació de les valls d'Espot i de Boí*. (Institut d'Estudis Catalans, Barcelona, 1992).

52. Banc de dades de la biodiversitat de Catalunya.

53. GBIF occurrence download. (2021).

54. Smith, G. L. *A flora of the Tahoe basin and neighboring areas and supplement*. (University of San Francisco, San Francisco, 1984).

55. Jump, A. S., Huang, T.-J. & Chou, C.-H. Rapid altitudinal migration of mountain plants in Taiwan and its implications for high altitude biodiversity. *Ecography* **35**, 204–210 (2012).

56. Nowak, A. *et al.* *Illustrated flora of Tajikistan and adjacent areas*. (Polish Academy of Sciences, Warsaw, 2020).

57. Natural Values Atlas.

58. Welsh, S. L., Atwood, N. D., Goodrich, S. & Higgins, L. C. *A Utah flora*. (Monte L. Bean Life Science Museum, Provo, 1993).

59. Hokche, O., Berry, P. E. & Huber, O. *Nuevo catálogo de la flora vascular de Venezuela*. (Caracas: Fundación Instituto Botánico de Venezuela, Caracas, 2008).

60. Bussmann, R. W. The forests of Mount Kenya (Kenya): vegetation, ecology, destruction and management of a tropical mountain forest ecosystem. (1994).

61. Mbuni, Y. M. *et al.* An annotated checklist of vascular plants of Cherangani hills, Western Kenya. *PhytoKeys* **120**, 1–90 (2019).

62. Fertig, W. F., Massatti, R. T., Nelson, B. E. & Hartman, R. L. Annotated checklist of the vascular flora of the Wind River range, Wyoming (U.S.A.). *Journal of the Botanical Research Institute of Texas* **7**, 905–939 (2013).

63. Gilchrist, G. W. Specialists and generalists in changing environments. I. Fitness landscapes of thermal sensitivity. *The American Naturalist* **146**, 252–270 (1995).
